# Supplementary material for: Mycobacterium tuberculosis SecA2-dependent activation of host Rig-I/MAVs signaling is not conserved in Mycobacterium marinum
Source: PLoS One. 2024 Feb 23;19(2):e0281564. doi: 10.1371/journal.pone.0281564 (PMC10889897; doi:10.1371/journal.pone.0281564)
Supplement: S1 Table — (PDF) [file pone.0281564.s001.pdf]

**Bacterial Strain**	**Genotype**	**Reference**
M. marinum M	Wild type parental strain	Patricia Champion; ATCC BAA535
ΔsecA2	*M. marinum* M with deletion of *secA2* (MMAR_2698)	This study
Δ*secA2/ pMOPs_secA2_MM*	*M. marinum* M Δ*secA2* deletion strain complemented with the *M. marinum* version of *secA2* (MMAR_2698) under control of the pMOP promoter.	This study
Δ*secA2/ pMOPs_secA2_MT*	*M. marinum* M Δ*secA2* deletion strain complemented with the *M. tuberculosis* version of *secA2* (Rv1821) under control of the pMOP promoter.	This study
Δ*esxBA*	*M. marinum* M with deletion of *ΔesxBA*	Eric Brown (Gao et al., 2004)

S1 Table: A list of bacterial strains used in this study.
