# Supplementary material for: Mycobacterium tuberculosis SecA2-dependent activation of host Rig-I/MAVs signaling is not conserved in Mycobacterium marinum
Source: PLoS One. 2024 Feb 23;19(2):e0281564. doi: 10.1371/journal.pone.0281564 (PMC10889897; doi:10.1371/journal.pone.0281564)
Supplement: S2 Table — All primers were purchased through Invitrogen. (PDF) [file pone.0281564.s002.pdf]

Name	Sequence 5'→3'	Application	Reference
secA2-A	cgtggtgtcacgctcgtCTATCGGGCGGCCATCTGTTTTC	Amplifies ~1.5kb upstream flanking region of MMAR_2698	This study
secA2-B	aaacgccgggcttaagCGCTGTACAACTGCGCACGCT		
secA2-C	ttgtacagcgcttaagCCCGGCGTTTTCCGGTGAGC	Amplifies ~1.5kb downstream flanking region of MMAR_2698	This study
secA2-D	gcagtcaggcaccgtGCTACTGGTGGGATTGTTGTCGAC		
secA2-X	GCCGTCGTCAACGTTCTCACC	Checks for the removal of *secA2* (MMAR_2698) from the *M. marinum* genome. Primers sit just outside the *secA2* ORF	This study
secA2-Y	GCTGAACATCAGAATCGCCACCG		
MMAR_2698 P1	aggagtccagccatGTGCGCAGTTGTACAGCGGCGC	Amplifies *M. marinum secA2* (MMAR_2698) open reading frame for insertion into the pMOP plasmid	This study
MMAR_2698 P2	gcctgagcggtcccgactagtTCACCGGAAAACGCCGGGCAGG		
Rv1821_P1	aggagtccagccatGTGAACGTGCACGGTTGTCCACG	Amplifies *M. tuberculosis secA2* (Rv1821) open reading frame for insertion into the pMOP plasmid	This study
Rv1821_P2	gcctgagcggtcccgactagtTCAGCGGAACACCCCGGGCAG		
sigA_F	TCGAGGTGATCAACAAGCTG	*M. marinum sigA* qPCR primers	Williams et al., 2017
sigA_R	TGGATCTCCAGCACCTTCTC		
secA2_qPCR_F	GACGAGTTTCACCGGATG	*M. marinum* and *M. tuberculosis secA2* qPCR primers	This study
secA2_qPCR_R	ATGTAGGTCCATGTCGACG		
OMF096	ACGAGCGTGACACCACGATGCC	Amplifies the p2NIL vector	Sanchez et al., 2020
OMF097	ACGGTGCCTGACTGCGTTAGCA		

	ATTTAACTG		
OMF057	CATATGGCTGGACTCCTGAATTCTGCAGCTG	Amplifies the pMOP vector	Sanchez et al., 2020
OMF630	ACTAGTCGGGACCGCTCAGGCGTCC		

S2 Table: A list of oligonucleotide primers used throughout this paper. All primers were purchased through Invitrogen.
