# Supplementary material for: Mycobacterium tuberculosis SecA2-dependent activation of host Rig-I/MAVs signaling is not conserved in Mycobacterium marinum
Source: PLoS One. 2024 Feb 23;19(2):e0281564. doi: 10.1371/journal.pone.0281564 (PMC10889897; doi:10.1371/journal.pone.0281564)
Supplement: S3 Table — (PDF) [file pone.0281564.s003.pdf]

Plasmid name	Genotype	Reference
p2NIL	Parental vector for allelic exchange; *kan*^{R}, *amp*^{R}, *oriE*	Parish & Stoker, 2000
pGOAL	Amp^R Parental vector for allelic exchange; GOAL cassette includes hyg^R, lacZ, sacB, oriE	
p2NIL_Δ*MMAR_2698_GOAL*	*M. marinum secA2* flanking regions (MMAR_2698); *kan*^{R}, *hyg*^{R}, *lacZ, sacB*, *oriE*	This study
pMH406	pMOP parental plasmid; hyg^R	Jeffery Cox;<br>Reyna et al., 2016
pMOP_*MMAR_2698*	*M. marinum secA2* (*MMAR_2698*) behind the pMOP promoter; *hyg*^{R}, *attB* integration	This study
pMOP_Rv1821	*M. tuberculosis secA2* (*Rv1821*) behind the pMOP promoter; *hyg*^{R}, *attB* integration	This study

S3 Table: A list of plasmids used in this study.
