# Supplementary material for: Mycobacterium tuberculosis SecA2-dependent activation of host Rig-I/MAVs signaling is not conserved in Mycobacterium marinum
Source: PLoS One. 2024 Feb 23;19(2):e0281564. doi: 10.1371/journal.pone.0281564 (PMC10889897; doi:10.1371/journal.pone.0281564)
Supplement: S4 Table — (PDF) [file pone.0281564.s004.pdf]

Ab name	Specificity	Clone	Ab type	MW (kDa)	Titer	Source
α-SecA2	SecA2 (Rv1821)	-	Rabbit Polyclonal	90	1:10k	Miriam Braunstein; Guo et al., 2007
α-Rv0440	GroEL2 (Rv0440)	IT-56 (CBA1)	Mouse Monoclonal	65	1:10k	BEI Resources
α-MAVS	MAVS	E8Z7M	Rabbit Monoclonal	75	1:500	Cell Signaling Technology
α-β-actin	β-actin	13E5	Rabbit Monoclonal	45	1:500	Cell Signaling Technology
Goat α-Rabbit HRP	Rabbit IgG (H+L)	-	-	-	1:10k	Thermo Fischer Scientific
Goat α-Mouse HRP	Mouse IgG (H+L)	-	-	-	1:10k	Thermo Fischer Scientific

S4 Table: Primary and secondary antibodies used for western blot analysis in this study.
