# Supplementary material for: Mycobacterium tuberculosis SecA2-dependent activation of host Rig-I/MAVs signaling is not conserved in Mycobacterium marinum
Source: PLoS One. 2024 Feb 23;19(2):e0281564. doi: 10.1371/journal.pone.0281564 (PMC10889897; doi:10.1371/journal.pone.0281564)
Supplement: S1 Fig — Sequence alignment of M. marinum M (accession no.: ACC41141.1) and M. tuberculosis (QNF05793.1) SecA2. Sequences were obtained from NCBI and aligned using Clustal Omega 1.2.4. Conservation in amino acid sequence is denoted by an asterix (*) while changes resulting in strongly, weakly, or no similar chemical properties are denoted by a colon (:), period (.), or left blank. (PDF) [file pone.0281564.s005.pdf]

```
CLUSTAL O(1.2.4) multiple sequence alignment


Rv1821          MNVHGCPRIAACRCTDTHPRGRPAFAYRWFVPKTTRAQPGRLSSRFWRLLGASTEKNRSR 60
MMAR_2698       --MRSCTAAPGVSRTNTHARRGLGVAYRWAVSKTTRAQSGHLSSRFWRLLGATTEKNQNR 58
                  ::.*    .   *:** *   ..**** * ****** *:***********:****:.*

Rv1821          SLADVTASAEYDKEAADLSDEKLRKAAGLLNLDDLAESADIPQFLAIAREAAERRTGLRP 120
MMAR_2698       SLAQVTASADFDKEAADLNDEKLRKAAGLLNLEDLADSADIPQFLAIAREAGERATGLRP 118
                ***:*****::*******.*************:***:**************.** *****

Rv1821          FDVQLLGALRMLAGDVIEMATGEGKTLAGAIAAAGYALAGRHVHVVTINDYLARRDAEWM 180
MMAR_2698       FDVQLLGALRMLAGDVIEMATGEGKTLAGAIAAAGYALGGRHVHVVTINDYLARRDAEWM 178
                **************************************.*********************

Rv1821          GPLLDAMGLTVGWITADSTPDERRTAYDRDVTYASVNEIGFDVLRDQLVTDVNDLVSPNP 240
MMAR_2698       APLLEAMDLTVGWITAESTGADRRAAYECDVTYASVNEIGFDVLRDQLVTDVADLVSPNP 238
                .***:**.********:**  :**:**: *********************** *******

Rv1821          DVALIDEADSVLVDEALVPLVLAGTTHRETPRLEIIRLVAELVGDKDADEYFATDSDNRN 300
MMAR_2698       DVALIDEADSVLVDEALVPLVLAGTTHRETPRLEIIKLVGQLVKDKDADEYFATDADSRN 298
                ************************************:**.:** ***********:*.**

Rv1821          VHLTEHGARKVEKALGGIDLYSEEHVGTTLTEVNVALHAHVLLQRDVHYIVRDDAVHLIN 360
MMAR_2698       VHLTEAGARKVEKALGGIDLYSEEHVGTTLTEVNVALHAHVLLQRDVHYIVRDDAVHLIN 358
                ***** ******************************************************

Rv1821          ASRGRIAQLQRWPDGLQAAVEAKEGIETTETGEVLDTITVQALINRYATVCGMTGTALAA 420
MMAR_2698       ASRGRIAQLQRWPDGLQAAVEAKEGIETTETGEVLDTITVQALINRYVTVCGMTGTALAA 418
                ***********************************************.************

Rv1821          GEQLRQFYQLGVSPIPPNKPNIREDEADRVYITTAAKNDGIVEHITEVHQRGQPVLVGTR 480
MMAR_2698       GEQLRQFYKLGVSPIPPNTPNIREDESDRVYITAAAKNDAIVEHIAEVHDTGQPVLVGTR 478
                ********:*********.*******:******:*****.*****:***: *********

Rv1821          DVAESEELHERLVRRGVPAVVLNAKNDAEEARVIAEAGKYGAVTVSTQMAGRGTDIRLGG 540
MMAR_2698       DVAESEDLHERLLRRDIPAVVLNAKNDAEEAAVIAEAGTLSRVTVSTQMAGRGTDIRLGG 538
                ******:*****:**.:************** ******. . ******************

Rv1821          SDEADHDRVAELGGLHVVGTGRHHTERLDNQLRGRAGRQGDPGSSVFFSSWEDDVVAANL 600
MMAR_2698       SDEADHDQVAELGGLHVVGTGRHHTQRLDNQLRGRAGRQGDPGSSVFFSSWEDDVVAANL 598
                *******:*****************:**********************************

Rv1821          DHNKLPMATDENGRIVSPRTGSLLDHAQRVAEGRLLDVHANTWRYNQLIAQQRAIIVERR 660
MMAR_2698       DGNKLPMETDEDGQIVSAKAAGLLDHAQRVAEGRMLDVHANTWRYNQLIAQQRAIIVDRR 658
                * ***** ***:*:*** ::..************:**********************:**

Rv1821          NTLLRTVTAREELAELAPKRYEELSDKVSEERLETICRQIMLYHLDRGWADHLAYLADIR 720
MMAR_2698       NTLLRTATAREELADLAPKRYKELSETVSEDRLEKICRMIMLYHLDRGWADHLAYLADIR 718
                ******.*******:******:***:.***:***.*** *********************

Rv1821          ESIHLRALGRQNPLDEFHRMAVDAFASLAADAIEAAQQTFETANVLDHEPGLDLSKLARP 780
MMAR_2698       ESIHLRALGRQNPLDEFHRMAVDAFASLAADAIEAAQQTFETANVLEDEPGLDLSKLARP 778
                **********************************************:.************

Rv1821          TSTWTYMVNDNPLSDDTLSALSLPGVFR    808
MMAR_2698       TSTWTYMVNDNPLSDDTLSTLSLPGVFR    806
                *******************:********
```

S5 Fig: *M. marium* and *M. tuberculosis* SecA2 shares 88.34% sequence identity. Sequence alignment of *M. marinum* M (accession no.: ACC41141.1) and *M. tuberculosis* (QNF05793.1) SecA2. Sequences were obtained from NCBI and aligned using Clustal Omega 1.2.4. Conservation in amino acid sequence is denoted by an asterix (*) while changes resulting in strongly, weakly, or no similar chemical properties are denoted by a colon (:), period (.), or left blank.
