# Supplementary material for: Mycobacterium tuberculosis SecA2-dependent activation of host Rig-I/MAVs signaling is not conserved in Mycobacterium marinum
Source: PLoS One. 2024 Feb 23;19(2):e0281564. doi: 10.1371/journal.pone.0281564 (PMC10889897; doi:10.1371/journal.pone.0281564)
Supplement: S2 Fig — Primer pairs X & Y were used to amplify secA2 from its endogenous location in the genome in the ΔsecA2 M. marinum strain. Purified PCR products were sequenced by the Genomics Core at the University of Notre Dame using the secA2-X primer. Deletion of secA2 was confirmed using the chromatogram viewer FinchTV version 1.5.0. Arrows above the chromatogram indicate the primer sequences (secA2-B and secA2-C) and encoded AflII restriction site used in the generation of the knockout. (PDF) [file pone.0281564.s006.pdf]

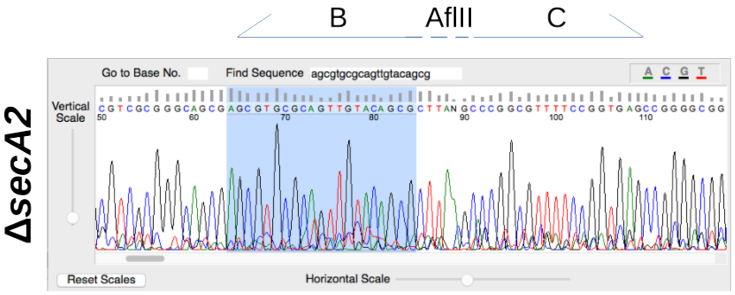

S6 Fig: Confirmation of the ΔsecA2 *M. marinum* strain by Sanger sequencing. Primer pairs X & Y were used to amplify *secA2* from its endogenous location in the genome in the ΔsecA2 *M. marinum* strain. Purified PCR products were sequenced by the Genomics Core at the University of Notre Dame using the secA2-X primer. Deletion of *secA2* was confirmed using the chromatogram viewer FinchTV version 1.5.0. Arrows above the chromatogram indicate the primer sequences (secA2-B and secA2-C) and encoded AflII restriction site used in the generation of the knockout.
