# Supplementary material for: Mycobacterium tuberculosis SecA2-dependent activation of host Rig-I/MAVs signaling is not conserved in Mycobacterium marinum
Source: PLoS One. 2024 Feb 23;19(2):e0281564. doi: 10.1371/journal.pone.0281564 (PMC10889897; doi:10.1371/journal.pone.0281564)
Supplement: S3 Fig — Representative images from three independent biological replicate growth curves. A logistic growth curve model was fit to average bacterial growth optical density (OD600) values (from three technical replicates each measured in technical triplicate). Individual plot titles represent the bacterial strain, biological replicate number, and growth rate (r). Best fit line is indicated in red. (PDF) [file pone.0281564.s007.pdf]

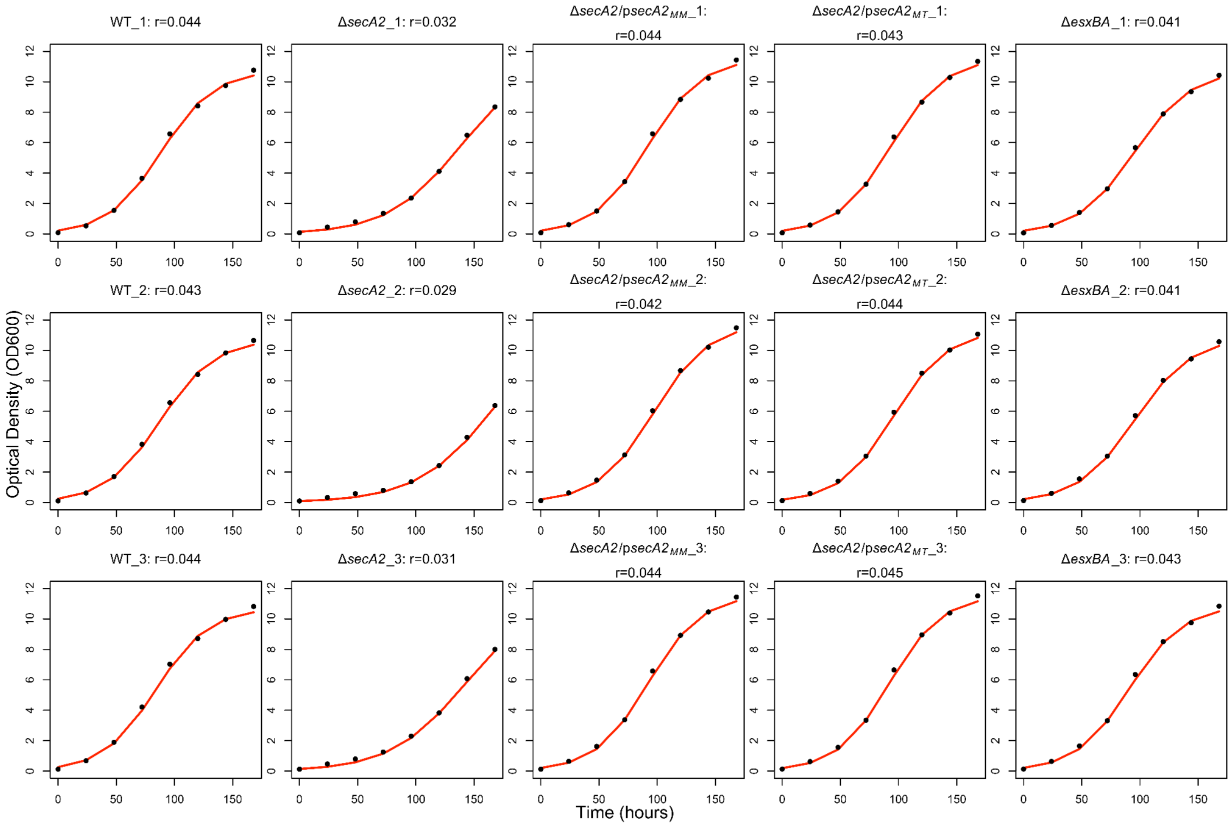

S7 Fig: **Δ*secA2 M. marinum* has a lower growth rate in nutrient rich media than WT and complemented strains**. Representative images from three independent biological replicate growth curves. A logistic growth curve model was fit to average bacterial growth optical density (OD600) values (from three technical replicates each measured in technical triplicate). Individual plot titles represent the bacterial strain, biological replicate number, and growth rate (r). Best fit line is indicated in red.
