# Supplementary material for: Mycobacterium tuberculosis SecA2-dependent activation of host Rig-I/MAVs signaling is not conserved in Mycobacterium marinum
Source: PLoS One. 2024 Feb 23;19(2):e0281564. doi: 10.1371/journal.pone.0281564 (PMC10889897; doi:10.1371/journal.pone.0281564)
Supplement: S4 Fig — The resulting parameter outputs (A; representative of the average for each biological replicate) were used to compare bacterial growth rates (r). These growth rates were plotted (B), with points representative of growth rates for each technical replicate (n = 3) for each biological replicate (n = 3) for a total of 9 data points per stain. Bar heights represent the average value of all 9 data points. Statistical significance was assessed using a one-way ANOVA followed by a Dunnett’s pairwise comparison relative to WT. *** p<0.001. (PDF) [file pone.0281564.s008.pdf]

A.

sample	k	n0	r
WT_1	10,7274913949484	0,21520786361925	0,0440056023552211
Δ*secA2*_1	11,565285549338	0,135773588014184	0,0319953167930311
Δ*secA2*/p*secA2*$_{MM}$_1	11,5162066566508	0,206892908825466	0,0435266447197418
Δ*secA2*/p*secA2*$_{MT}$_1	11,5494539959183	0,199322371271335	0,0431952869204296
Δ*esxBA*_1	10,7257858024095	0,204611102524859	0,0413698640093804
WT_2	10,7047628387631	0,244440537873155	0,0428589553769309
Δ*secA2*_2	13,3766725673294	0,0881688551241262	0,0291628240833739
Δ*secA2*/p*secA2*$_{MM}$_2	11,7517079236676	0,197576124882229	0,0420724250117277
Δ*secA2*/p*secA2*$_{MT}$_2	11,2819238227648	0,17464134173783	0,0435123490753641
Δ*esxBA*_2	10,8276384890036	0,212834322118197	0,0409279470793812
WT_3	10,699011840645	0,257832960239918	0,0440755720908912
Δ*secA2*_3	11,529900793971	0,133874853823205	0,0311044156978132
Δ*secA2*/p*secA2*$_{MM}$_3	11,5733708400361	0,197883078052666	0,0438605588013956
Δ*secA2*/p*secA2*$_{MT}$_3	11,5432351583446	0,187224660335566	0,0445246444696151
Δ*esxBA*_3	10,8737700945955	0,205254785870312	0,0433490734821979

B.

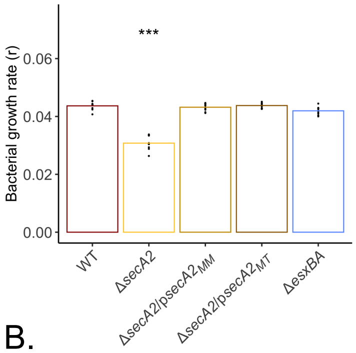

S8 Fig: Bacterial *in vitro* growth measurements were fit to a logarithmic growth curve using the Growthcurver R package. The resulting parameter outputs (A; representative of the average for each biological replicate) were used to compare bacterial growth rates (r). These growth rates were plotted (B), with points representative of growth rates for each technical replicate (n=3) for each biological replicate (n=3) for a total of 9 data points per stain. Bar heights represent the average value of all 9 data points. Statistical significance was assessed using a one-way ANOVA followed by a Dunnett's pairwise comparison relative to WT. *** p<0.001.
