# Supplementary material for: Mycobacterium tuberculosis SecA2-dependent activation of host Rig-I/MAVs signaling is not conserved in Mycobacterium marinum
Source: PLoS One. 2024 Feb 23;19(2):e0281564. doi: 10.1371/journal.pone.0281564 (PMC10889897; doi:10.1371/journal.pone.0281564)
Supplement: S5 Fig — M. marinum strains were grown to exponential phase in 7H9 supplemented with 10% OADC and 0.2% tyloxapol before being subcultured into fresh media at an optical density (OD600) of 0.05. Bacterial growth was monitored by measuring the OD600 every 24-hours for 7 days. (PDF) [file pone.0281564.s009.pdf]

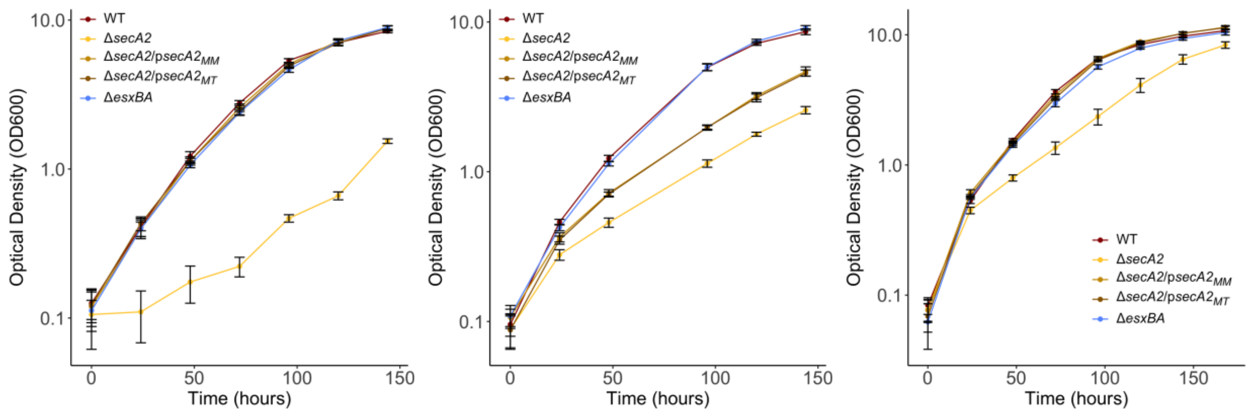

S9 Fig: Variation is Δ*secA2* growth in different batches of OADC. *M. marinum* strains were grown to exponential phase in 7H9 supplemented with 10% OADC and 0.2% tyloxapol before being subcultured into fresh media at an optical density (OD_{600}) of 0.05. Bacterial growth was monitored by measuring the OD_{600} every 24-hours for 7 days.
