# Supplementary material for: Mycobacterium tuberculosis SecA2-dependent activation of host Rig-I/MAVs signaling is not conserved in Mycobacterium marinum
Source: PLoS One. 2024 Feb 23;19(2):e0281564. doi: 10.1371/journal.pone.0281564 (PMC10889897; doi:10.1371/journal.pone.0281564)
Supplement: S6 Fig — M. marinum strains were grown to exponential phase in 7H9 supplemented with 0.1% tween80 before being subcultured into fresh media at an optical density (OD600) of 0.05. Upon subculturing, a pronounced aggregation phenotype was observed. Δ8 is shorthand for ΔsecA2. (PDF) [file pone.0281564.s010.pdf]

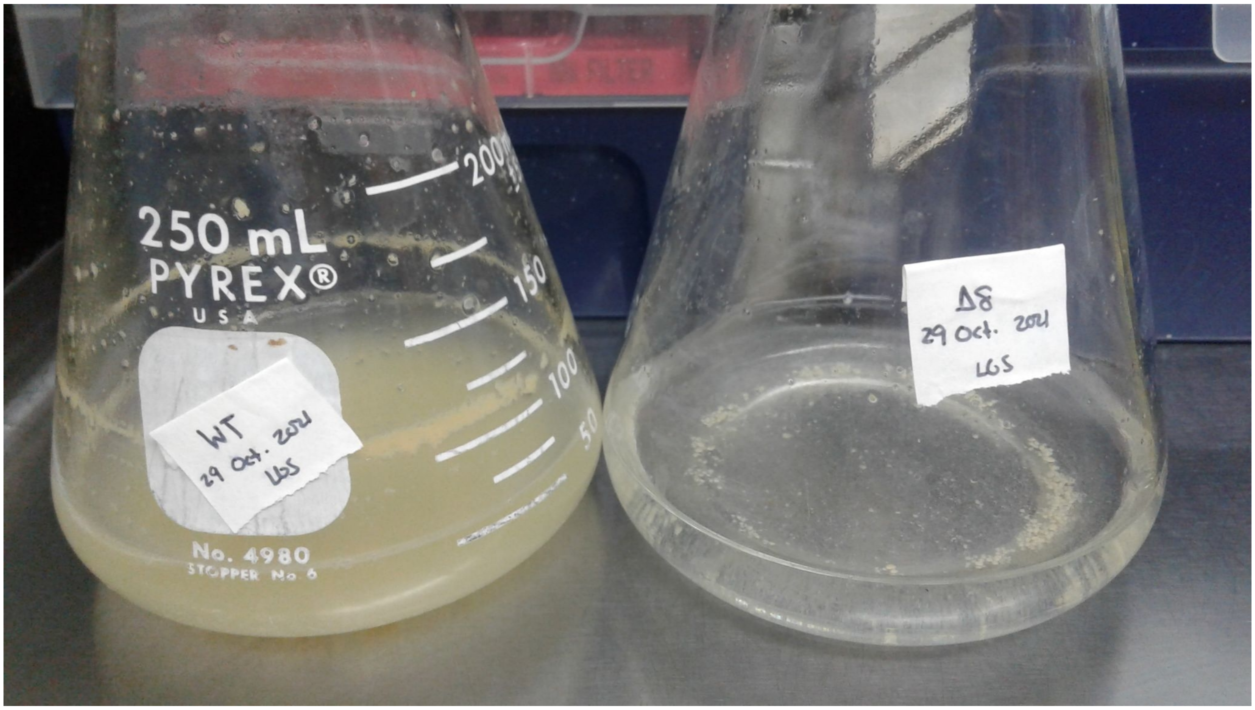

S10 Fig: Δ*secA2* bacteria aggregate in the presence of minimal media. *M. marinum* strains were grown to exponential phase in 7H9 supplemented with 0.1% tween80 before being subcultured into fresh media at an optical density (OD_{600}) of 0.05. Upon subculturing, a pronounced aggregation phenotype was observed. Δ8 is shorthand for Δ*secA2*.
