# Supplementary material for: Mycobacterium tuberculosis SecA2-dependent activation of host Rig-I/MAVs signaling is not conserved in Mycobacterium marinum
Source: PLoS One. 2024 Feb 23;19(2):e0281564. doi: 10.1371/journal.pone.0281564 (PMC10889897; doi:10.1371/journal.pone.0281564)
Supplement: S7 Fig — M. marinum strains were grown to exponential phase in 7H9 supplemented with 10% OADC and 0.2% tyloxapol before being subcultured into fresh media with and without 0.05% SDS at an OD600 of 0.8. Twenty-four hours later, these cultures were serially diluted and plated onto 7H11 agar plates supplemented with 10% OADC in technical triplicate. Image is representative of two biological replicates each plated in technical triplicate (A). Colony forming units were quantified from agars plates following incubation at 32°C with 5% CO2 for one week and bacterial growth in SDS was quantified relative to growth in nutrient rich media lacking SDS. Inset represents the same data with an adjusted y-axis (B). Statistical significance was calculated using the non-parametric Kruskal-Wallis test followed by pairwise comparison with a Wilcoxon Rank Sum test relative to M. marinum ΔsecA2. Δ8 = ΔsecA2; *** p-values ≤ 0.001. (PDF) [file pone.0281564.s011.pdf]

A.

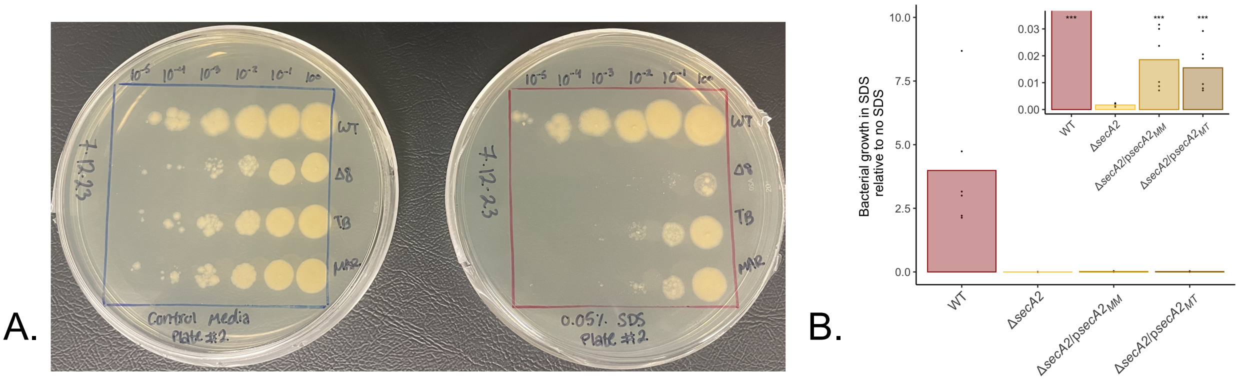

B.

S11 Fig*: M. marinum ΔsecA2 strain is sensitivity to SDS* *M. marinum* strains were grown to exponential phase in 7H9 supplemented with 10% OADC and 0.2% tyloxapol before being subcultured into fresh media with and without 0.05% SDS at an OD600 of 0.8. Twenty-four hours later, these cultures were serially diluted and plated onto 7H11 agar plates supplemented with 10% OADC in technical triplicate. Image is representative of two biological replicates each plated in technical triplicate (A). Colony forming units were quantified from agars plates following incubation at 32 with 5% CO_2 for one week and bacterial growth in SDS was quantified relative to growth in nutrient rich media lacking SDS. Inset represents the same data with an adjusted y-axis (B). Statistical significance was calculated using the non-parametric Kruskal-Wallis test followed by pairwise comparison with a Wilcoxon Rank Sum test relative to *M. marinum* Δ*secA2*. *Δ8 = ΔsecA2;* *** p-values ≤ 0.001.
