# Supplementary material for: Mycobacterium tuberculosis SecA2-dependent activation of host Rig-I/MAVs signaling is not conserved in Mycobacterium marinum
Source: PLoS One. 2024 Feb 23;19(2):e0281564. doi: 10.1371/journal.pone.0281564 (PMC10889897; doi:10.1371/journal.pone.0281564)
Supplement: S8 Fig — At 2, 48, and 96-hours post infection, macrophage monolayers were washed three times and lysed in sterile water containing 0.2% tyloxapol. Whole cell lysate dilutions were plated in technical triplicate and grown at 32°C for 1 week. Colony forming units were counted and linear equations were fit to log transformed colony counts (red line) for each of the three biological replicates. Individual plot titles represent the bacterial strain, biological replicate number, and best fit line equation for average data from each biological replicate. (PDF) [file pone.0281564.s012.pdf]

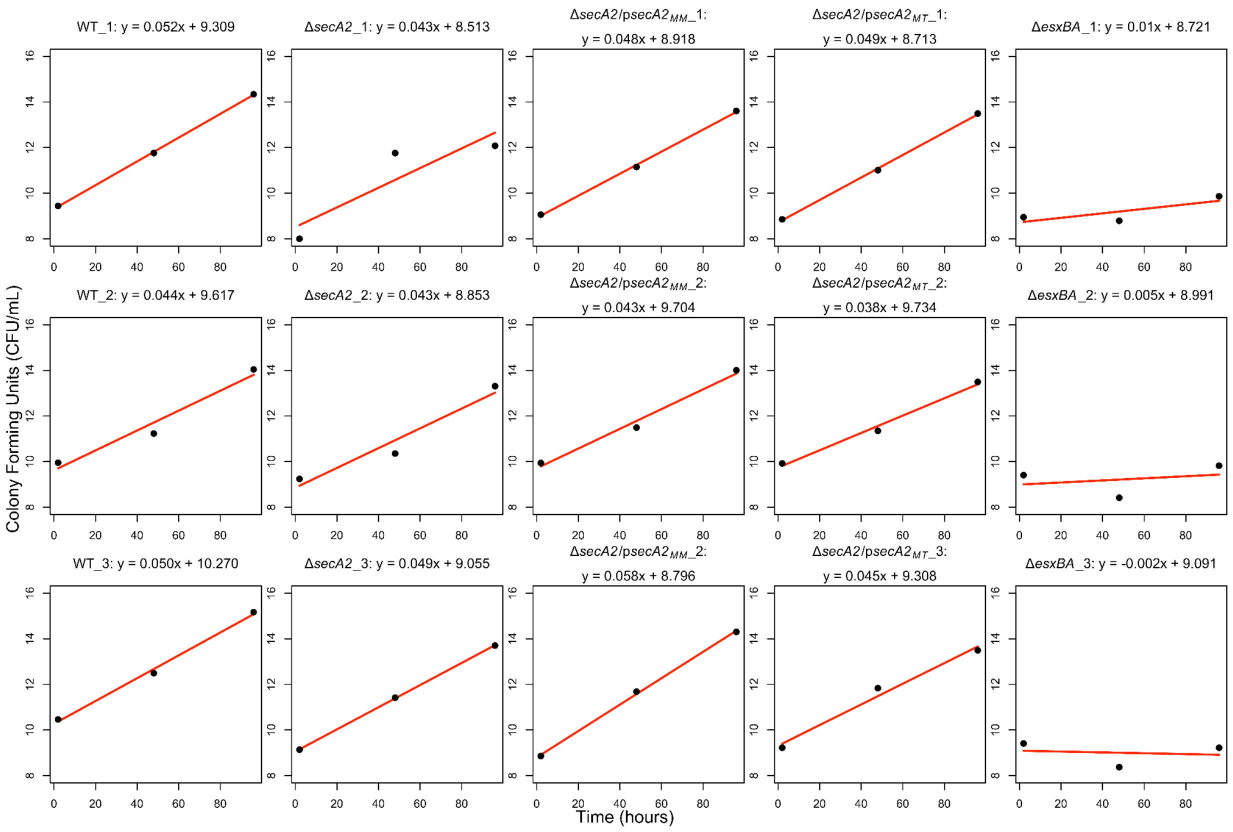

S12 Fig: Triplicate wells of WT BMDMs were infected with *M. marinum* strains at an MOI of 0.2. At 2, 48, and 96-hours post infection, macrophage monolayers were washed three times and lysed in sterile water containing 0.2% tyloxapol. Whole cell lysate dilutions were plated in technical triplicate and grown at 32°C for 1 week. Colony forming units were counted and linear equations were fit to log transformed colony counts (red line) for each of the three biological replicates. Individual plot titles represent the bacterial strain, biological replicate number, and best fit line equation for average data from each biological replicate.
