# Supplementary material for: Mycobacterium tuberculosis SecA2-dependent activation of host Rig-I/MAVs signaling is not conserved in Mycobacterium marinum
Source: PLoS One. 2024 Feb 23;19(2):e0281564. doi: 10.1371/journal.pone.0281564 (PMC10889897; doi:10.1371/journal.pone.0281564)
Supplement: S9 Fig — The resulting parameter outputs (A; representative of the average for each biological replicate) were used to compare bacterial growth rates (r). These growth rates were plotted (B), with points representative of growth rates for each biological replicate (n = 3). Bar heights represent the average value of all 9 data points. Statistical significance was assessed using a one-way ANOVA followed by a Dunnett’s pairwise comparison relative to WT. *** p<0.001. (PDF) [file pone.0281564.s013.pdf]

A.

sample	intercept	slope (growth rate, r)
WT_1	9.30913	0.05215
Δ*secA2*_1	8.51347	0.04314
Δ*secA2*/p*secA2*$_{MM}$_1	8.91777	0.04839
Δ*secA2*/p*secA2*$_{MT}$_1	8.7133	0.0494
Δ*esxBA*_1	8.721205	0.009847
WT_2	9.61741	0.04364
Δ*secA2*_2	8.85334	0.04342
Δ*secA2*/p*secA2*$_{MM}$_2	9.70387	0.04331
Δ*secA2*/p*secA2*$_{MT}$_2	9.73448	0.03806
Δ*esxBA*_2	8.991189	0.004569
WT_3	10.2696	0.0501
Δ*secA2*_3	9.05527	0.04859
Δ*secA2*/p*secA2*$_{MM}$_3	8.79575	0.05793
Δ*secA2*/p*secA2*$_{MT}$_3	9.30843	0.04535
Δ*esxBA*_3	9.090988	-0.001855

B.

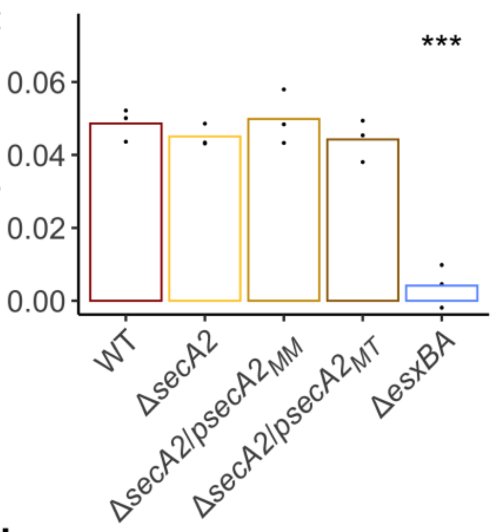

S13 Fig: Bacterial intracellular growth colony counts were fit to a linear regression model. The resulting parameter outputs (A; representative of the average for each biological replicate) were used to compare bacterial growth rates (r). These growth rates were plotted (B), with points representative of growth rates for each biological replicate (n=3). Bar heights represent the average value of all 9 data points. Statistical significance was assessed using a one-way ANOVA followed by a Dunnett’s pairwise comparison relative to WT. *** p<0.001.
