# Supplementary material for: Mycobacterium tuberculosis SecA2-dependent activation of host Rig-I/MAVs signaling is not conserved in Mycobacterium marinum
Source: PLoS One. 2024 Feb 23;19(2):e0281564. doi: 10.1371/journal.pone.0281564 (PMC10889897; doi:10.1371/journal.pone.0281564)
Supplement: S10 Fig — Statistical significance was assessed using a one-way ANOVA followed by a Dunnett’s pairwise comparison relative to WT. * p<0.05. (PDF) [file pone.0281564.s014.pdf]

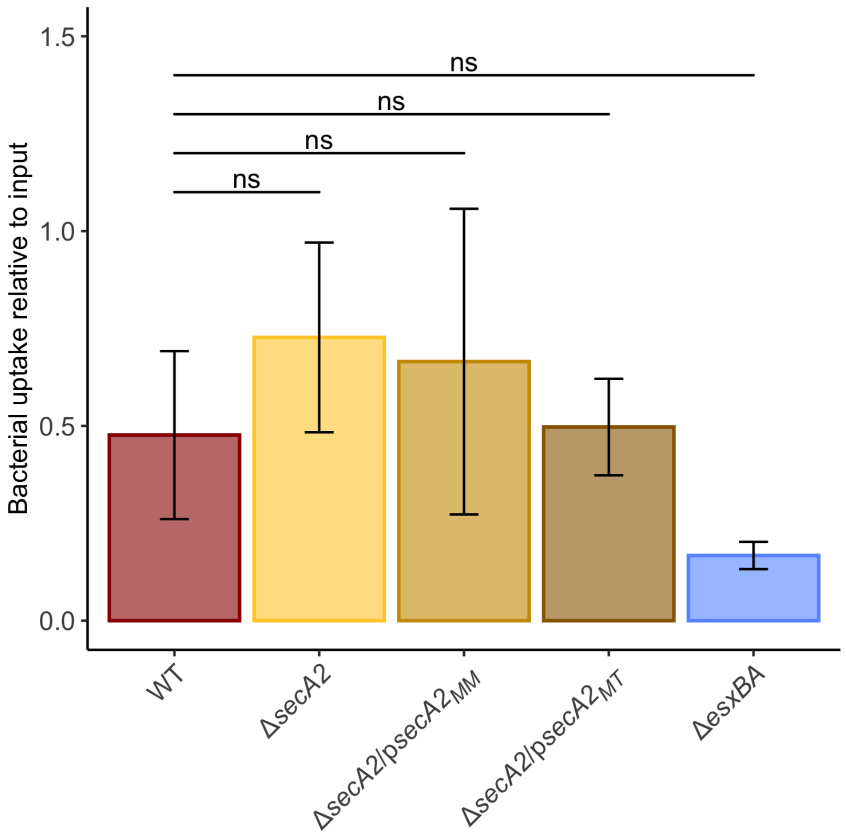

S14 Fig: The number of bacterial colony forming units present at 2hpi (uptake) relative to the number of bacteria added to each well at the start of the infection (input). Statistical significance was assessed using a one-way ANOVA followed by a Dunnett’s pairwise comparison relative to WT. * $p<0.05$.
