# Supplementary material for: Mycobacterium tuberculosis SecA2-dependent activation of host Rig-I/MAVs signaling is not conserved in Mycobacterium marinum
Source: PLoS One. 2024 Feb 23;19(2):e0281564. doi: 10.1371/journal.pone.0281564 (PMC10889897; doi:10.1371/journal.pone.0281564)
Supplement: S11 Fig — To evaluate the IFN-β response from wild type BMDMs when levels of intracellular bacteria are added in increasing amounts, log-phase M. marinum cultures were used to infect wild type BMDMs at various MOIs. Following a 2 hour infection, macrophage monolayers were washed three times with PBS before being lysed and plated onto 7H11 + 10% agar plates in technical triplicate (A) or returned to the incubator in fresh media until twenty-four hours post infection. At twenty-four hours, macrophage culture supernatants were removed and examined for secreted levels of IFN-β by ELISA (B). To account for variability in the number of bacteria present at 2hpi, IFN-β levels were normalized to a standard number of bacteria (C). IFN-β Heat killed WT M. marinum was added to macrophages at an MOI of 10 as a control for bacterial cell lysis. Data is representative of 10 biological replicates. Statistical significance was calculated using the non-parametric Kruskal-Wallis test followed by pairwise comparison with a Wilcoxon Rank Sum test relative to WT. Statistical significance was defined as p-values ≤ 0.05; ***p<0.001, **p<0.01, *p<0.05, ns = not significant (p>0.05). All infections were conducted at and MOI of 1 unless otherwise noted. (PDF) [file pone.0281564.s015.pdf]

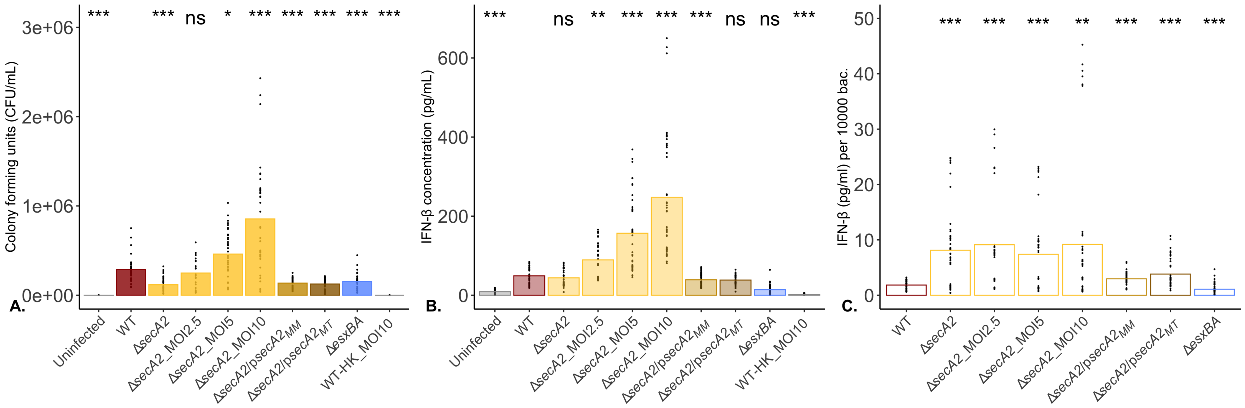

S15 Fig: WT BMDMs secrete increasing concentrations of IFN-β relative to increased MOIs. To evaluate the IFN-β response from wild type BMDMs when levels of intracellular bacteria are added in increasing amounts, log-phase *M. marinum* cultures were used to infect wild type BMDMs at various MOIs. Following a 2 hour infection, macrophage monolayers were washed three times with PBS before being lysed and plated onto 7H11 + 10% agar plates in technical triplicate (A) or returned to the incubator in fresh media until twenty-four hours post infection. At twenty-four hours, macrophage culture supernatants were removed and examined for secreted levels of IFN-β by ELISA (B). To account for variability in the number of bacteria present at 2hpi, IFN-β levels were normalized to a standard number of bacteria (C). IFN-β Heat killed WT *M. marinum* was added to macrophages at an MOI of 10 as a control for bacterial cell lysis. Data is representative of 10 biological replicates. Statistical significance was calculated using the non-parametric Kruskal-Wallis test followed by pairwise comparison with a Wilcoxon Rank Sum test relative to WT. Statistical significance was defined as p-values ≤ 0.05; ***p<0.001, **p<0.01, *p<0.05, ns=not significant (p>0.05). All infections were conducted at and MOI of 1 unless otherwise noted.
