# Supplementary material for: Mycobacterium tuberculosis SecA2-dependent activation of host Rig-I/MAVs signaling is not conserved in Mycobacterium marinum
Source: PLoS One. 2024 Feb 23;19(2):e0281564. doi: 10.1371/journal.pone.0281564 (PMC10889897; doi:10.1371/journal.pone.0281564)
Supplement: S12 Fig — BMDMs were infected with increasing amounts of ΔsecA2 M. marinum (MOIs of 1, 2.5, 5, and 10). Average levels of secreted IFN-β present in the supernatant 24 hours after the start of the infection were plotted against average CFUs plated at the end of the infection period (2hpi). A linear model was applied to each replicate to determine a best fit line (red). Equations defining the best fit line and associated R2 value are provided above each graph. (PDF) [file pone.0281564.s016.pdf]

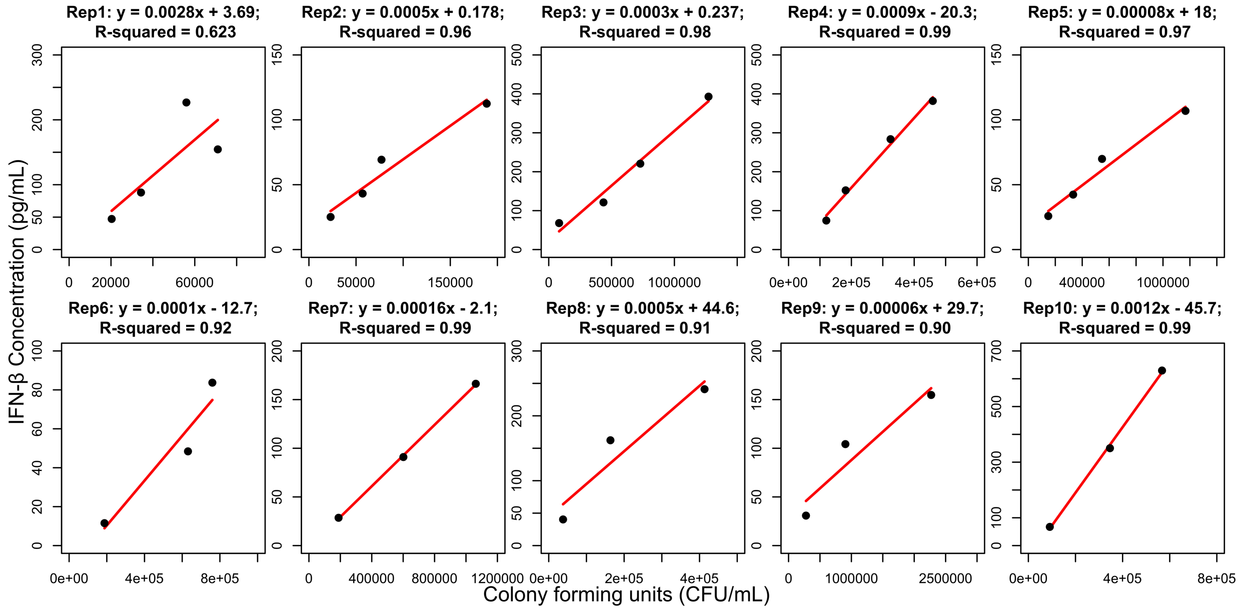

S16 Fig: Secreted levels of IFN-β increase with increasing Δ*secA2 M. marinum* MOI. BMDMs were infected with increasing amounts of Δ*secA2 M. marinum* (MOIs of 1, 2.5, 5, and 10). Average levels of secreted IFN-β present in the supernatant 24 hours after the start of the infection were plotted against average CFUs plated at the end of the infection period (2hpi). A linear model was applied to each replicate to determine a best fit line (red). Equations defining the best fit line and associated R^2 value are provided above each graph.
