# Supplementary material for: Mycobacterium tuberculosis SecA2-dependent activation of host Rig-I/MAVs signaling is not conserved in Mycobacterium marinum
Source: PLoS One. 2024 Feb 23;19(2):e0281564. doi: 10.1371/journal.pone.0281564 (PMC10889897; doi:10.1371/journal.pone.0281564)
Supplement: S13 Fig — RNA was isolated from BMDMs 8hpi with indicated M. marinum strains, purified, reverse transcribed, and examined for relative abundance of transcripts for IFN-β (left panel), Irf7 (middle panel), and Rig-I (right panel) relative to the housekeeping gene GAPDH for two biological replicates examined in technical duplicate. **p<0.01; all statistics were assessed relative to WT levels. (PDF) [file pone.0281564.s017.pdf]

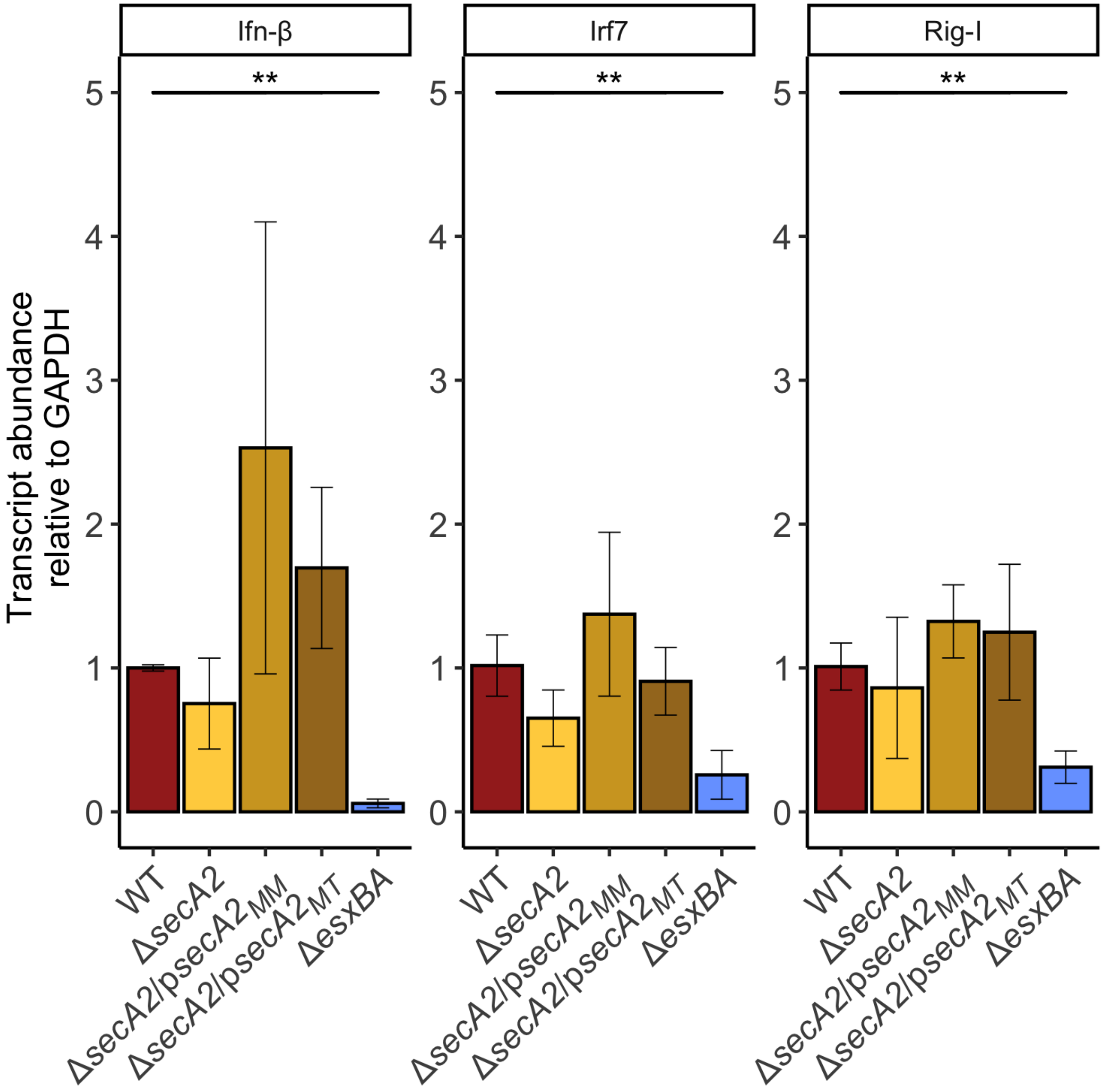

S17 Fig: Δ*secA2 M. marinum* induces similar levels of IFN-β, Irf7, and Rig-I as WT and complemented strains. RNA was isolated from BMDMs 8hpi with indicated *M. marinum* strains, purified, reverse transcribed, and examined for relative abundance of transcripts for IFN-β (left panel), Irf7 (middle panel), and Rig-I (right panel) relative to the housekeeping gene GAPDH for two biological replicates examined in technical duplicate. **$p<0.01$; all statistics were assessed relative to WT levels.
