# Supplementary material for: Mycobacterium tuberculosis SecA2-dependent activation of host Rig-I/MAVs signaling is not conserved in Mycobacterium marinum
Source: PLoS One. 2024 Feb 23;19(2):e0281564. doi: 10.1371/journal.pone.0281564 (PMC10889897; doi:10.1371/journal.pone.0281564)
Supplement: S14 Fig — Upon the removal of culture supernatants for ELISA analysis (Figs 5 and 6), macrophage monolayers from the same infections were lysed in 75ul RIPA lysis buffer. WCLs were quantified by BCA and 15 μg of protein were separated on an 8% SDS-PAGE gel. Proteins were transferred to a 0.45 μM, methanol-activated PVDF membrane and probed for Rig-I, MAVS, or β-actin (loading control). (PDF) [file pone.0281564.s018.pdf]

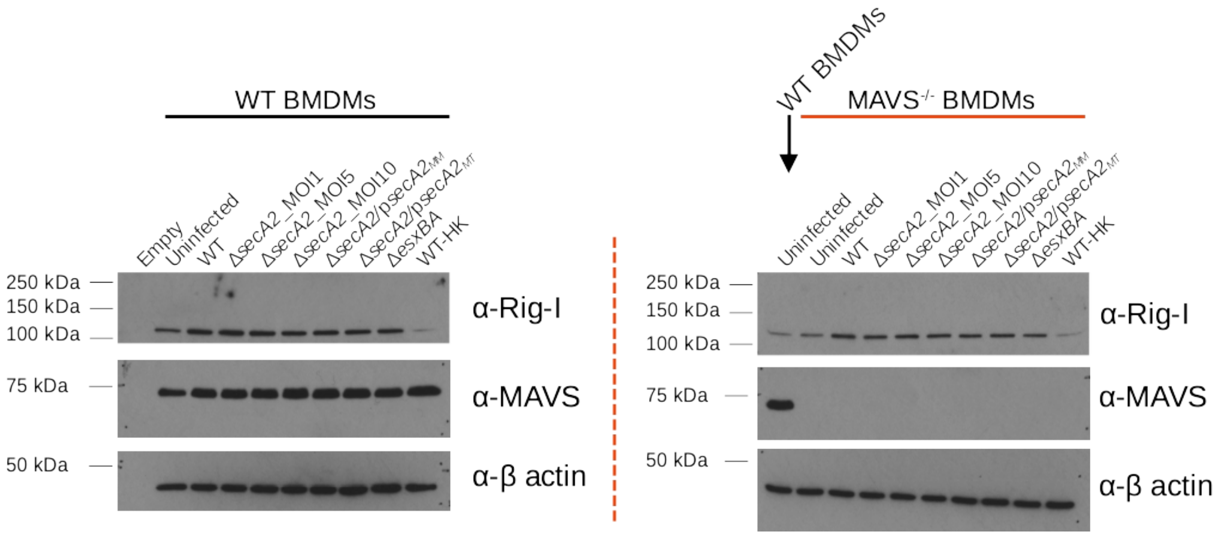

S18 Fig: Confirmation of MAVS knockout in MAVS$^{-/-}$ BMDMs. Upon the removal of culture supernatants for ELISA analysis (Fig 5 and Fig 6), macrophage monolayers from the same infections were lysed in 75ul RIPA lysis buffer. WCLs were quantified by BCA and 15 µg of protein were separated on an 8% SDS-PAGE gel. Proteins were transferred to a 0.45 µM, methanol-activated PVDF membrane and probed for Rig-I, MAVS, or β-actin (loading control).
