# Supplementary material for: Mycobacterium tuberculosis SecA2-dependent activation of host Rig-I/MAVs signaling is not conserved in Mycobacterium marinum
Source: PLoS One. 2024 Feb 23;19(2):e0281564. doi: 10.1371/journal.pone.0281564 (PMC10889897; doi:10.1371/journal.pone.0281564)

PCR images taken on a BioRad GelDoc 1000

Replicate 1: Bacterial WCLs generated 23-25 February2022 and diluted 1:50 in nuclease free water.

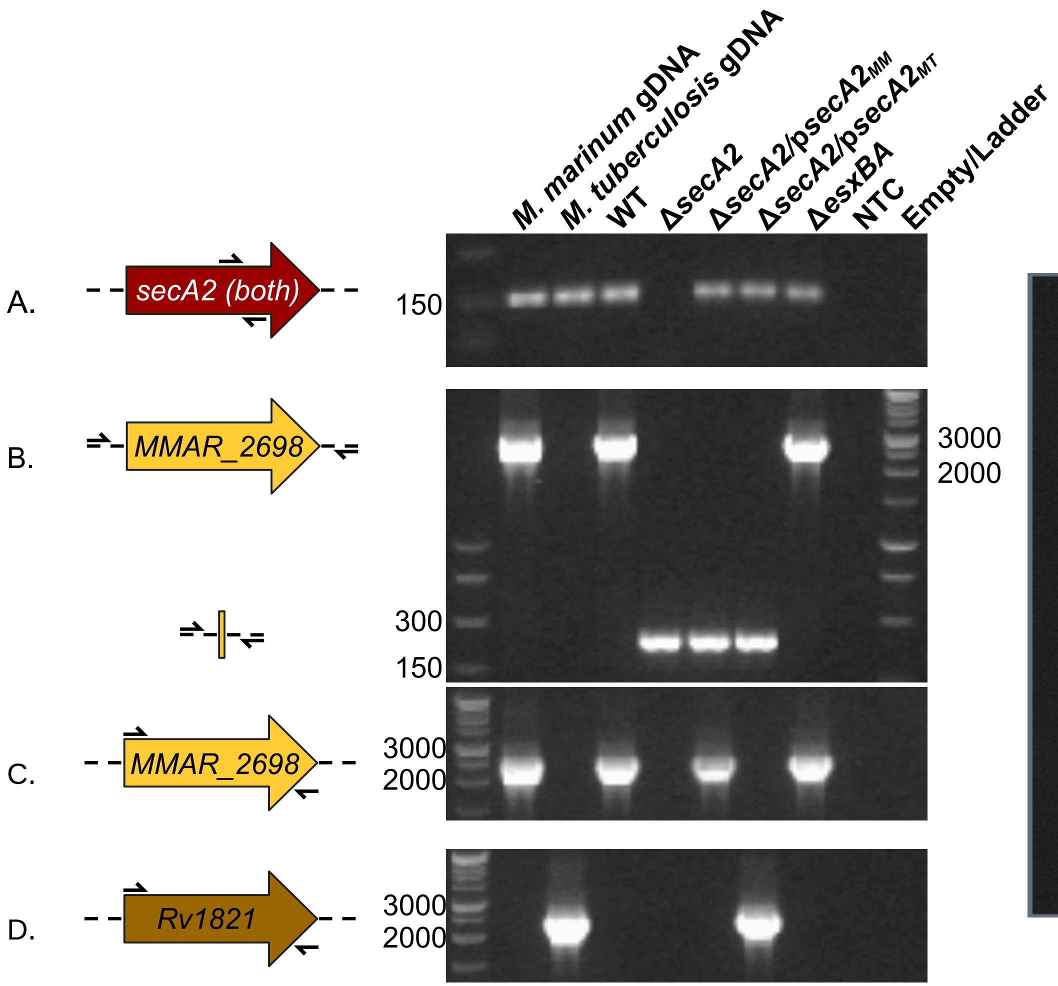

Original Image

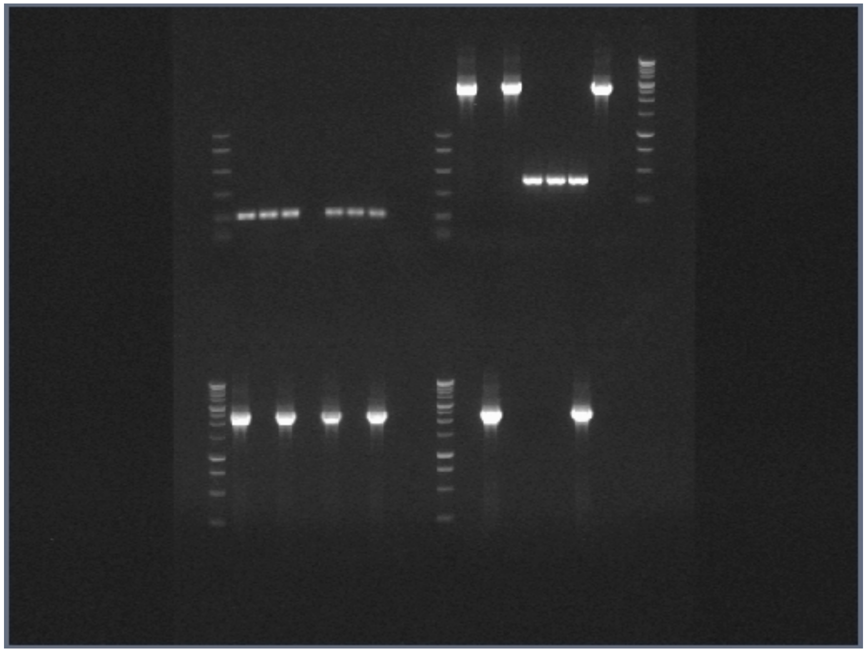

Replicate 2: Bacterial WCLs generated 16-18 March 2022 and diluted 1:50 in nuclease free water.

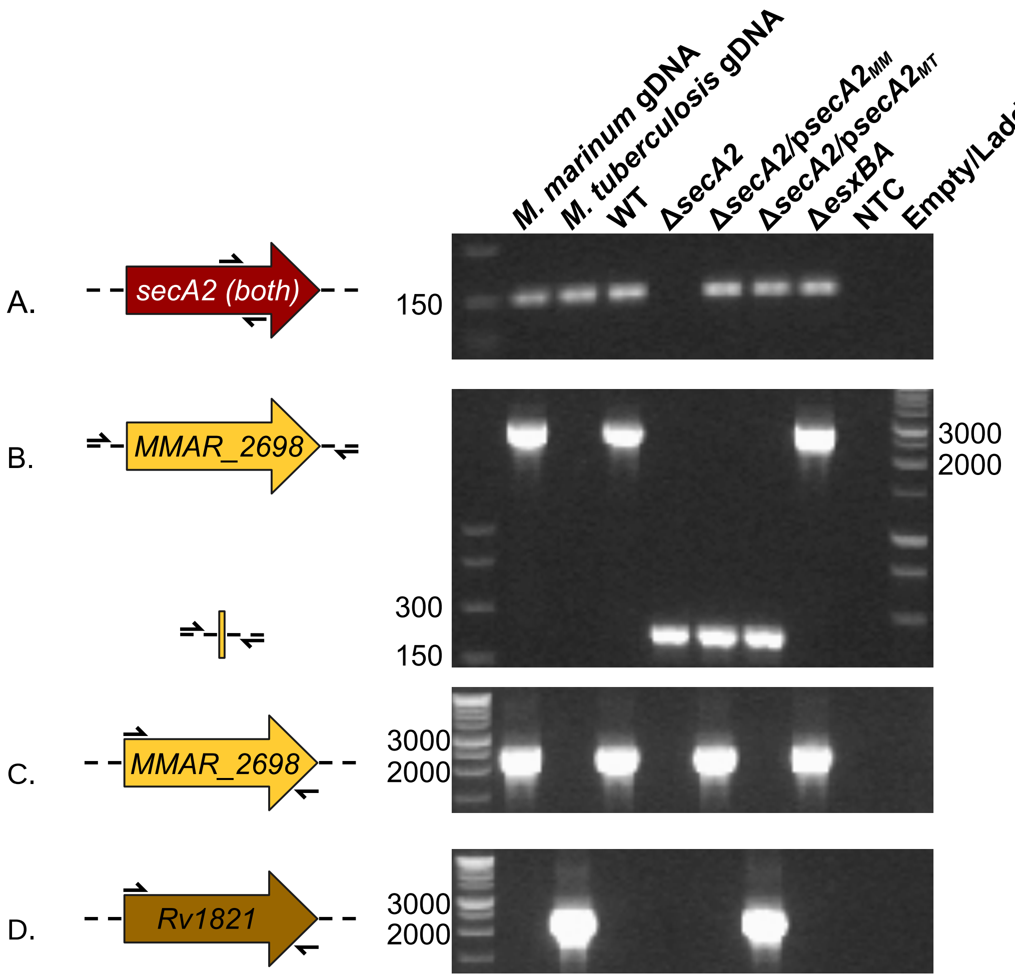

Original Image

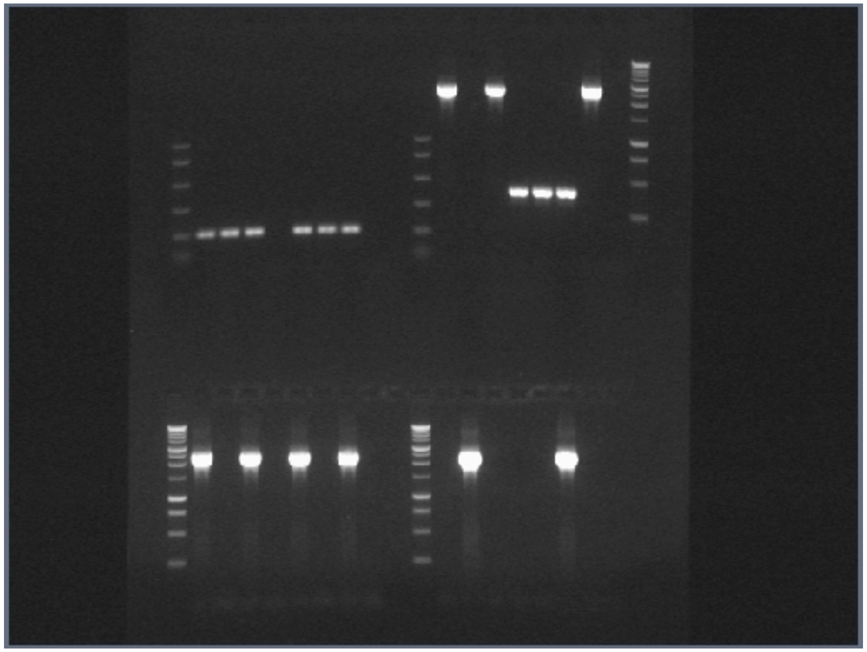

Replicate 3: Bacterial WCLs generated 23-24March 2022 and diluted 1:50 in nuclease free water.

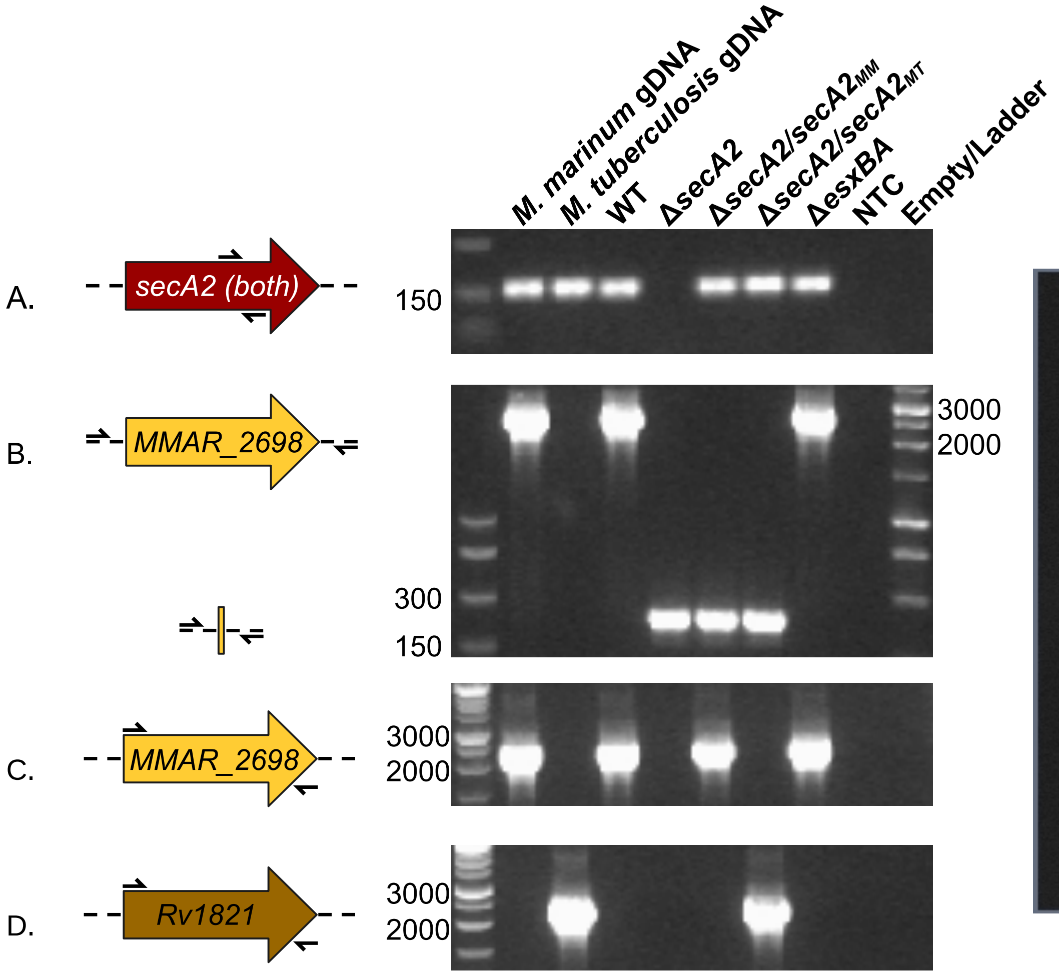

Original Image

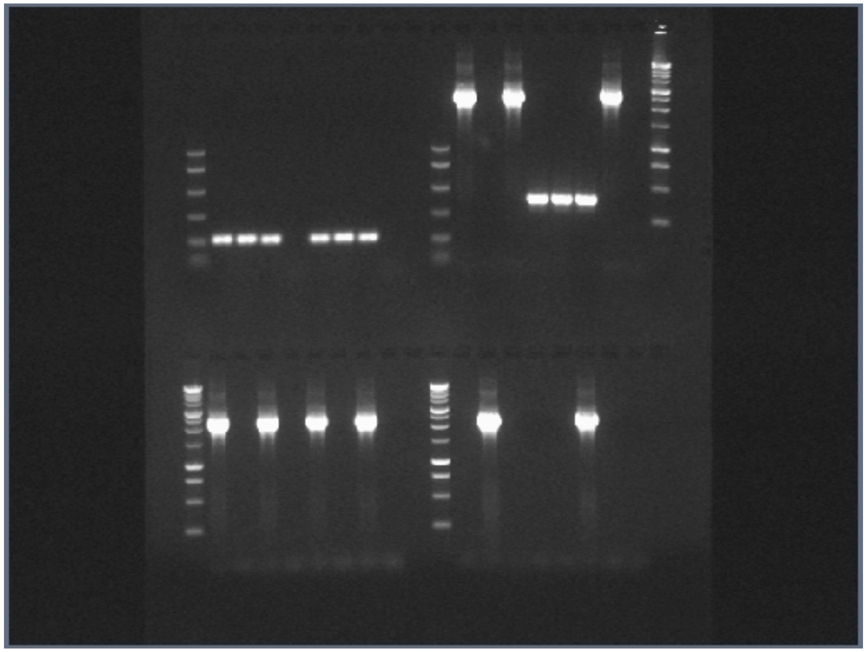

SecA2 and GroEL2 western blot scanned x-ray films

SecA2 and GroEL2_Rep. 1

Pellets from 23-25 Feb. 2022 *in vitro* growth curve

For each, entire membrane the exposure was increased by 25% and brightness by 100%.

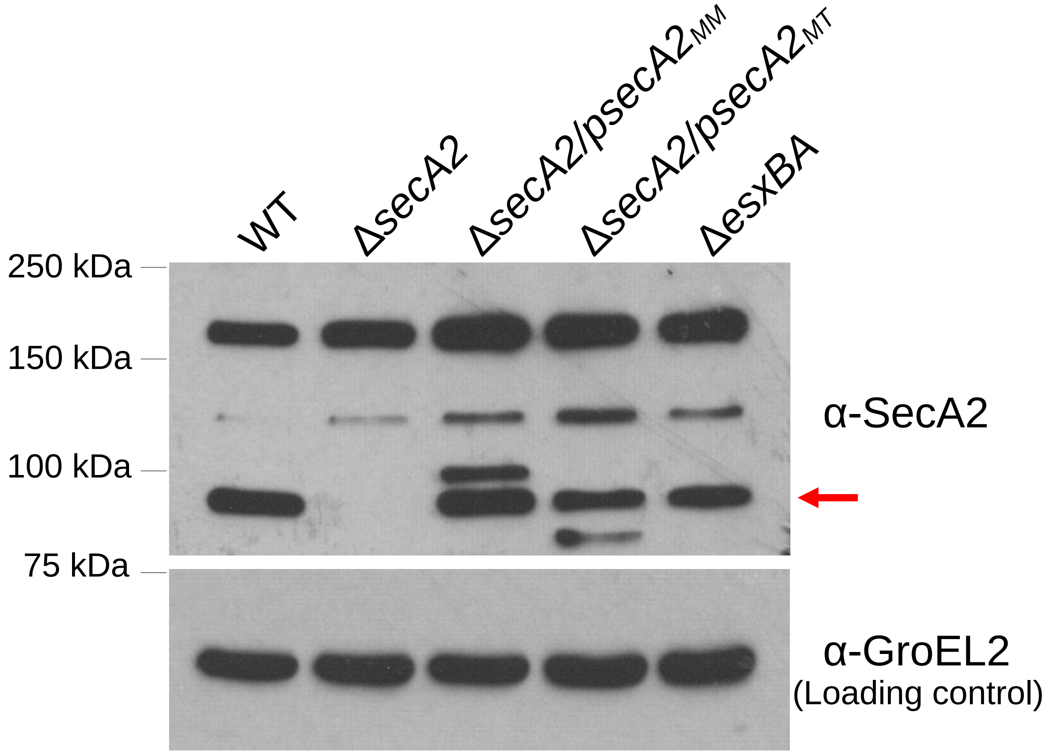

2 June 2022
From pellets 23 and 25 February 2022

Original Scan

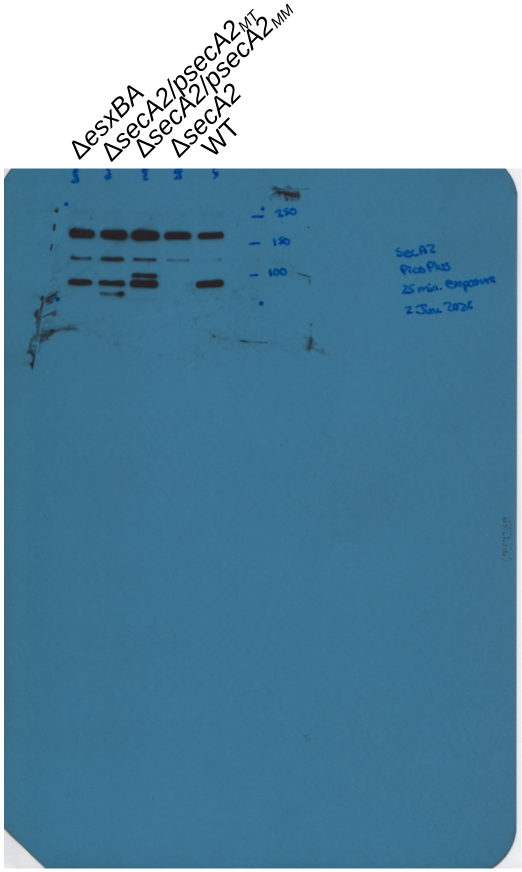

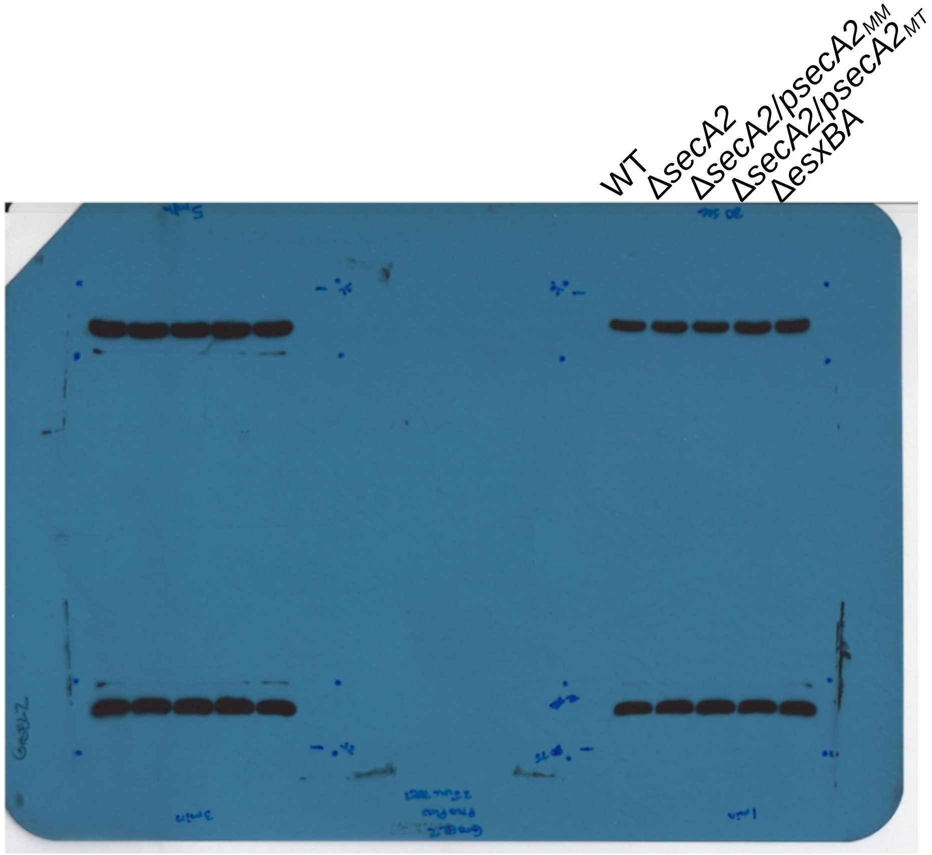

SecA2 and GroEL2_Rep. 2

Pellets from 16-18 March 2022 *in vitro* growth curve

For each, entire membrane the exposure was increased by 25% and brightness by 100%.

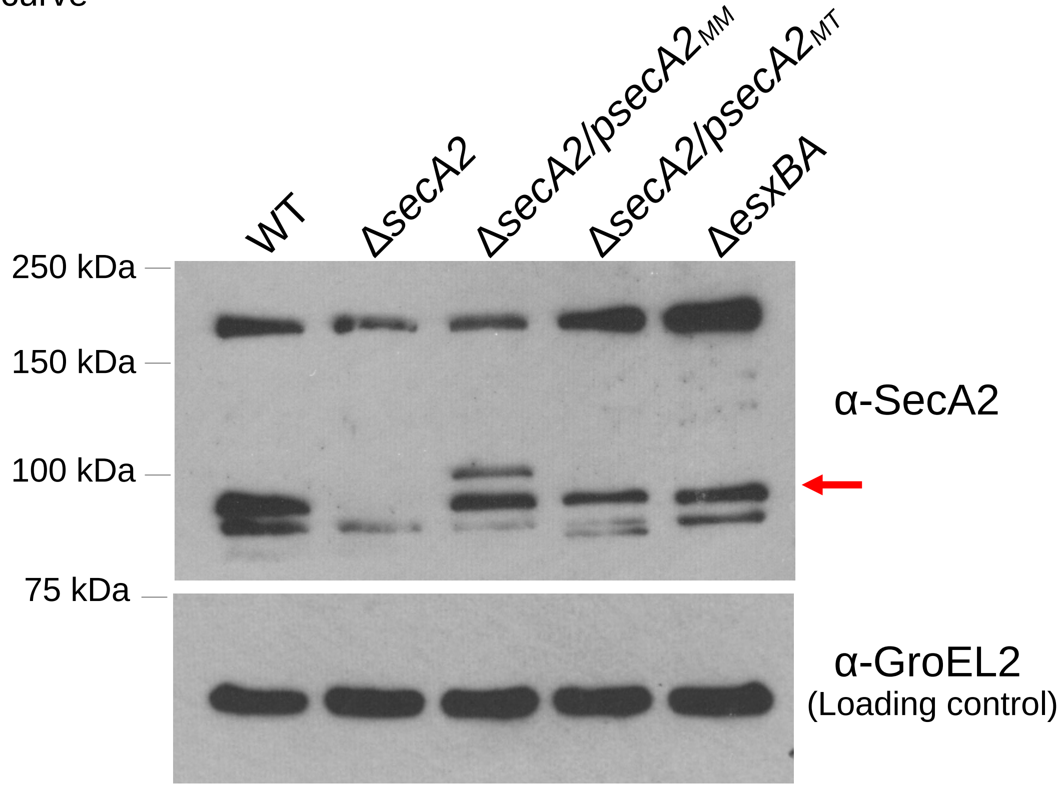

6 June 2022
From pellets 16 and 18 March 2022

Original Scan

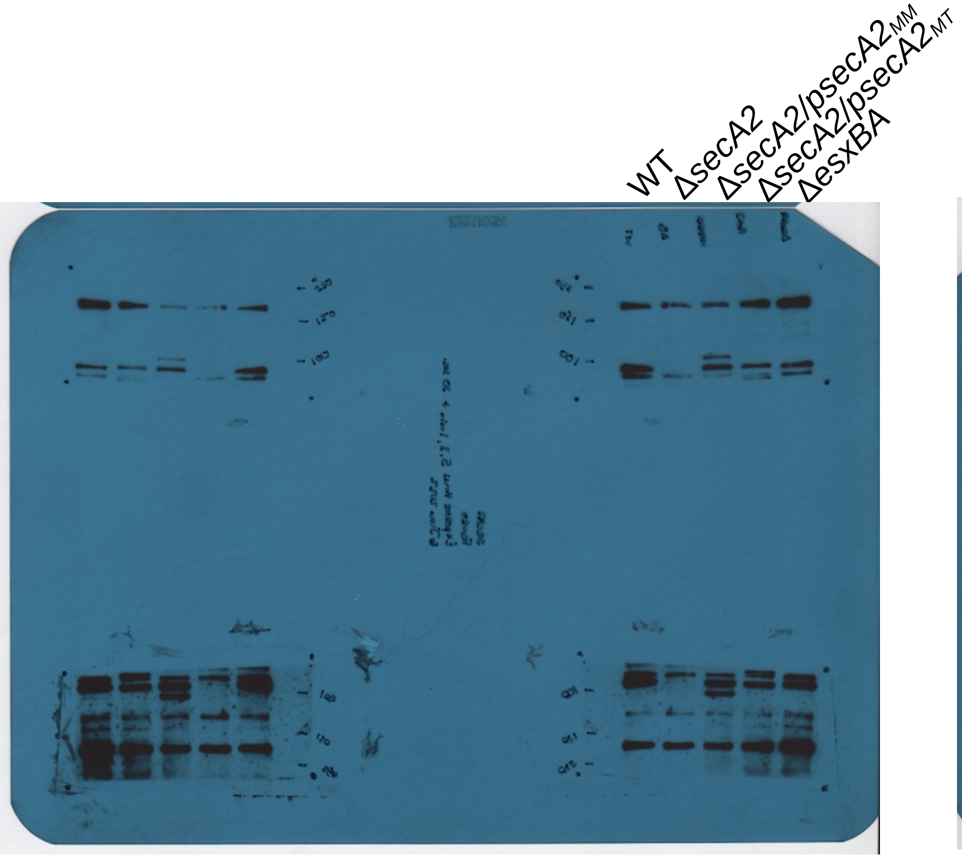

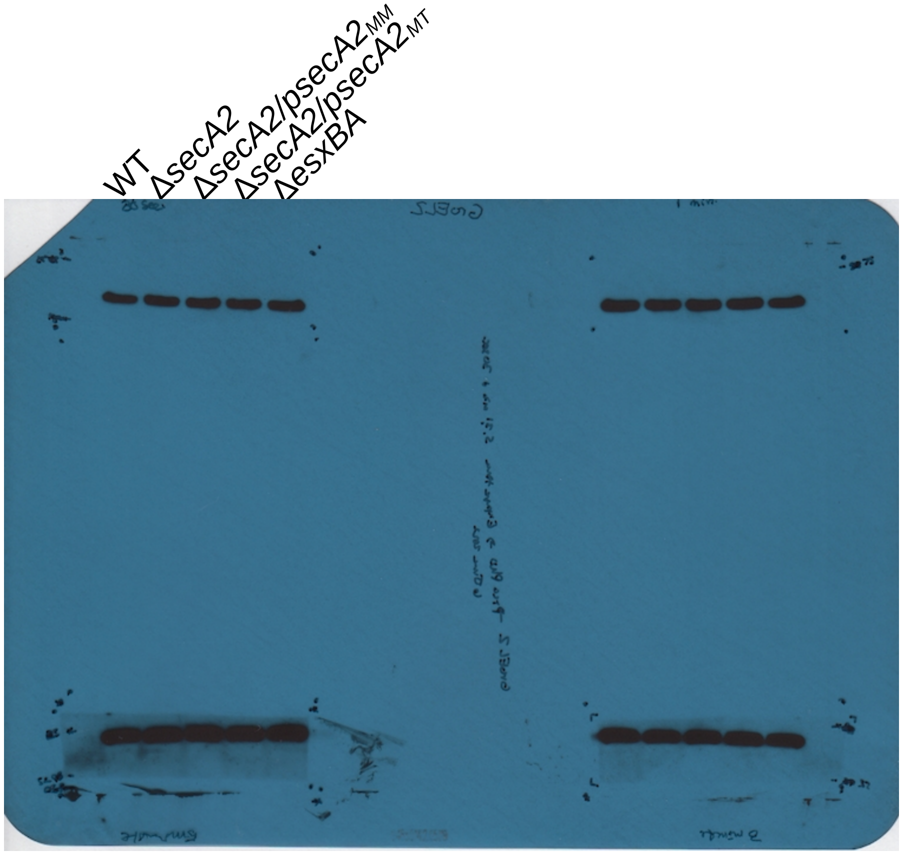

SecA2 and GroEL2_Rep. 3

Pellets from 23-24 March 2022 *in vitro* growth curve

For each, entire membrane the exposure was increased by 25% and brightness by 100%.

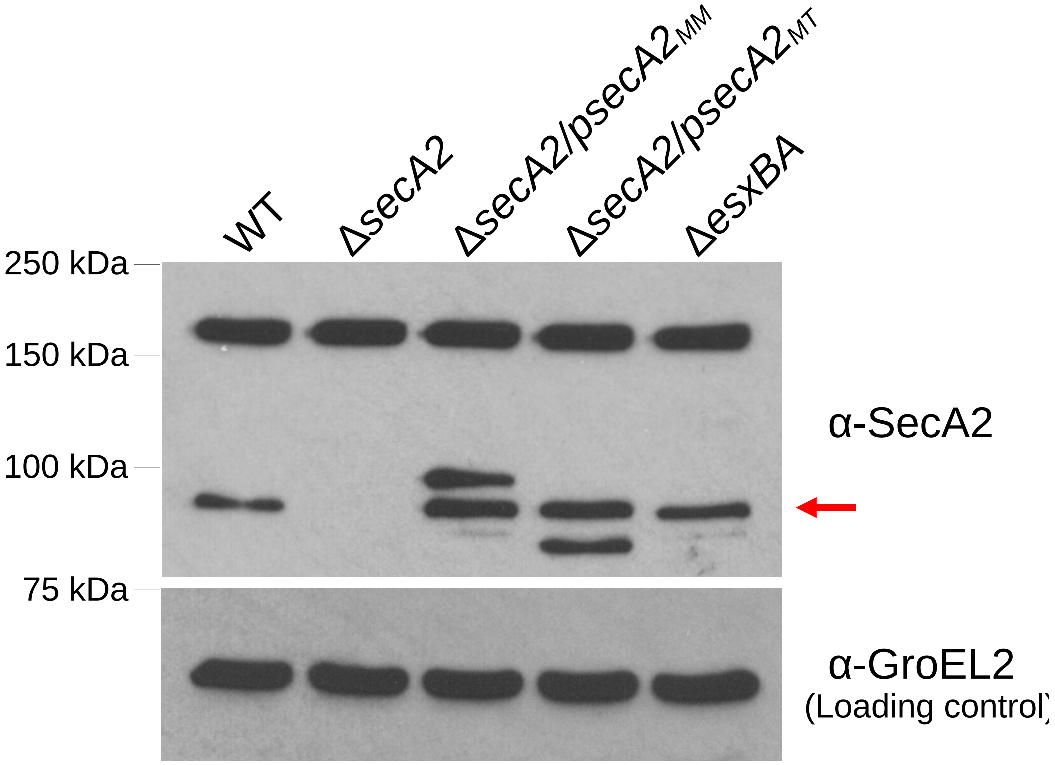

8 June 2022
From pellets 23 and 24 March 2022

Original Scan

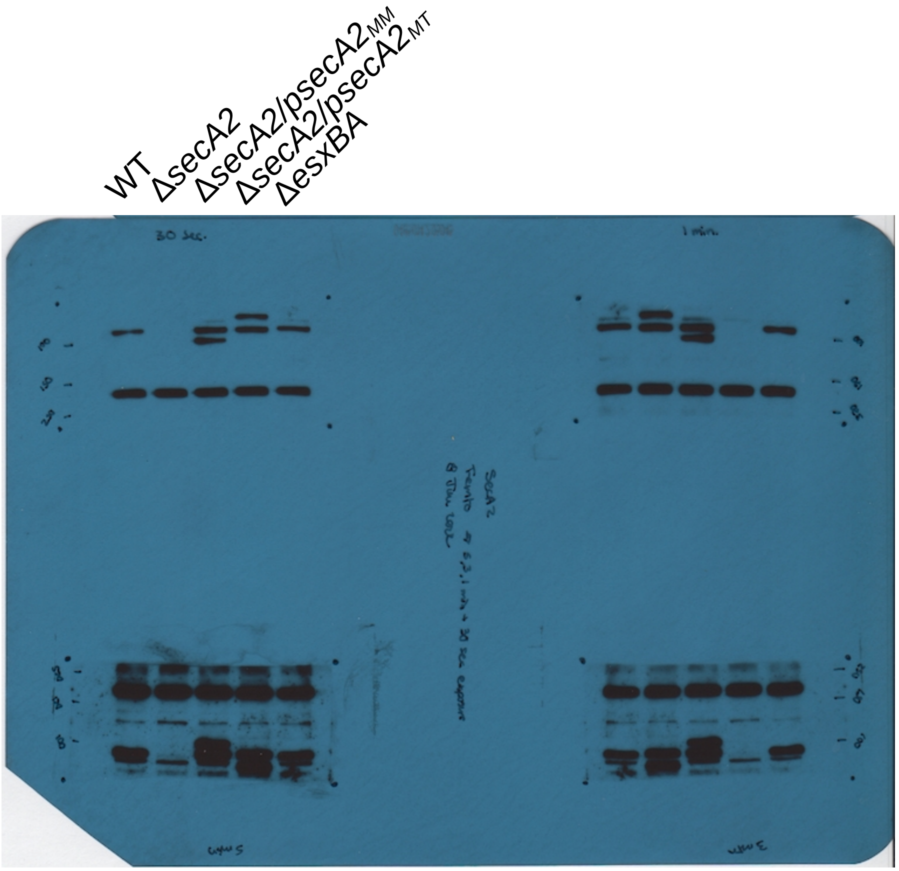

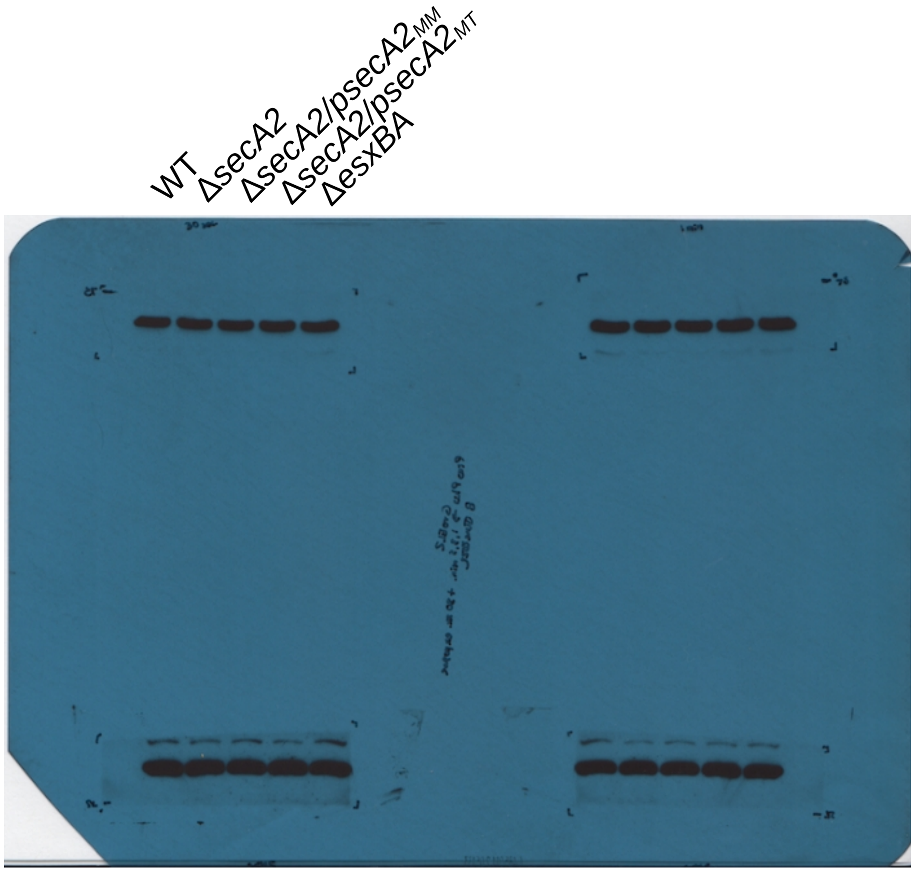

Supplement: S1 Raw images — (PDF) [file pone.0281564.s019.pdf]
